# Supplementary material for: A novel approach for measuring allostatic load highlights differences in stress burdens due to race, sex and smoking status
Source: PLoS One. 2025 Jun 2;20(5):e0323788. doi: 10.1371/journal.pone.0323788 (PMC12129187; doi:10.1371/journal.pone.0323788)
Supplement: S1 Table — For each biomarker, the table includes the calculated t-statistic, degrees of freedom (df), p-value. Significance levels are denoted as follows: ∎Represents significance between group (P < 0.1), * Represents significance between groups (p < 0.05), ** represents significance between groups (p < 0.01), *** represents significance between groups (p < 0.001), **** represents significance between groups (p < 0.0001). (DOCX) [file pone.0323788.s001.docx]

**S1 Table. T-test results for Individual Biomarker analysis between males and females.** For each biomarker, the table includes the calculated t-statistic, degrees of freedom (df), p-value. Significance levels are denoted as follows: ^∎^Represents significance between group (P < 0.1), * Represents significance between groups (p < 0.05), ** represents significance between groups (p < 0.01), *** represents significance between groups (p <0.001), **** represents significance between groups (p < 0.0001).

| **Biomarkers** | **Group 1** | **Group 2** | **n1** | **n2** | **Statistic** | **Degrees of Freedom (df)** | **p value** | **Significance** |
| --- | --- | --- | --- | --- | --- | --- | --- | --- |
| CRP | Males | Females | 23 | 40 | -3.490357552 | 43.2909912 | 0.00112 | ** |
| Cortisol | Males | Females | 23 | 40 | -1.100033076 | 59.84404953 | 0.276 | ns |
| Epinephrine | Males | Females | 19 | 33 | 0.84051153 | 22.44338768 | 0.409 | ns |
| Fibrinogen | Males | Females | 23 | 40 | -0.314792883 | 53.01164508 | 0.754 | ns |
| HDL | Males | Females | 23 | 40 | 0.060107168 | 59.51980385 | 0.952 | ns |
| Hba1c | Males | Females | 23 | 40 | 1.306316427 | 39.31540535 | 0.199 | ns |
| Noradrenaline | Males | Females | 23 | 40 | 3.062251711 | 46.78041361 | 0.00364 | ** |
