# Supplementary material for: A novel approach for measuring allostatic load highlights differences in stress burdens due to race, sex and smoking status
Source: PLoS One. 2025 Jun 2;20(5):e0323788. doi: 10.1371/journal.pone.0323788 (PMC12129187; doi:10.1371/journal.pone.0323788)
Supplement: Table S2 — For each biomarker, the table includes the calculated t-statistic, degrees of freedom (df), p-value. Significance levels are denoted as follows: ∎Represents significance between group (P < 0.1), * Represents significance between groups (p < 0.05), ** represents significance between groups (p < 0.01), *** represents significance between groups (p < 0.001), **** represents significance between groups (p < 0.0001). (DOCX) [file pone.0323788.s003.docx]

**S2 Table. T-test results for Individual Biomarker analysis between Black and White participants.** For each biomarker, the table includes the calculated t-statistic, degrees of freedom (df), p-value. Significance levels are denoted as follows: ^∎^Represents significance between group (P < 0.1), * Represents significance between groups (p < 0.05), ** represents significance between groups (p < 0.01), *** represents significance between groups (p <0.001), **** represents significance between groups (p < 0.0001).

| **Biomarkers** | **Group 1** | **Group 2** | **n1** | **n2** | **Statistic** | **Degrees of Freedom (df)** | **p value** | **Significance** |
| --- | --- | --- | --- | --- | --- | --- | --- | --- |
| CRP | Black | White | 31 | 32 | 1.361631499 | 54.85295189 | 0.179 | ns |
| Cortisol | Black | White | 31 | 32 | -0.843534028 | 44.53117052 | 0.403 | ns |
| Epinephrine | Black | White | 26 | 26 | -0.643738794 | 38.66863742 | 0.524 | ns |
| Fibrinogen | Black | White | 31 | 32 | 2.494101314 | 54.2248768 | 0.0157 | * |
| HDL | Black | White | 31 | 32 | -1.657528992 | 47.98524286 | 0.104 | ns |
| Hba1c | Black | White | 31 | 32 | 1.279432218 | 60.99752066 | 0.206 | ns |
| Noradrenaline | Black | White | 31 | 32 | -1.481140965 | 56.51351845 | 0.144 | ns |
