# Supplementary material for: A novel approach for measuring allostatic load highlights differences in stress burdens due to race, sex and smoking status
Source: PLoS One. 2025 Jun 2;20(5):e0323788. doi: 10.1371/journal.pone.0323788 (PMC12129187; doi:10.1371/journal.pone.0323788)
Supplement: S2 Fig — Significance levels are denoted as follows: ∎Represents significance between group (P < 0.1), * Represents significance between groups (p < 0.05), ** represents significance between groups (p < 0.01), *** represents significance between groups (p < 0.001), **** represents significance between groups (p < 0.0001). (DOCX) [file pone.0323788.s004.docx]

**S2 Fig. Boxplots for t-test results for Individual Biomarker analysis between Black and White participants.** Significance levels are denoted as follows: ^∎^Represents significance between group (P < 0.1), * Represents significance between groups (p < 0.05), ** represents significance between groups (p < 0.01), *** represents significance between groups (p <0.001), **** represents significance between groups (p < 0.0001).
