# Supplementary material for: A novel approach for measuring allostatic load highlights differences in stress burdens due to race, sex and smoking status
Source: PLoS One. 2025 Jun 2;20(5):e0323788. doi: 10.1371/journal.pone.0323788 (PMC12129187; doi:10.1371/journal.pone.0323788)
Supplement: S3 Table — For each biomarker, the table includes the calculated t-statistic, degrees of freedom (df), p-value. Significance levels are denoted as follows: ∎Represents significance between group (P < 0.1), * Represents significance between groups (p < 0.05), ** represents significance between groups (p < 0.01), *** represents significance between groups (p < 0.001), **** represents significance between groups (p < 0.0001). (DOCX) [file pone.0323788.s005.docx]

**S3 Table. T-test results for Individual Biomarker analysis between smokers and non-smokers.** For each biomarker, the table includes the calculated t-statistic, degrees of freedom (df), p-value. Significance levels are denoted as follows: ^∎^Represents significance between group (P < 0.1), * Represents significance between groups (p < 0.05), ** represents significance between groups (p < 0.01), *** represents significance between groups (p <0.001), **** represents significance between groups (p < 0.0001).

| **Biomarkers** | **Group 1** | **Group 2** | **n1** | **n2** | **Statistic** | **Degrees of Freedom (df)** | **p value** | **Significance** |
| --- | --- | --- | --- | --- | --- | --- | --- | --- |
| CRP | Non-Smokers | Cigarette Smokers | 30 | 33 | 1.288837037 | 46.9953227 | 0.204 | ns |
| Cortisol | Non-Smokers | Cigarette Smokers | 30 | 33 | 1.158371758 | 50.52470326 | 0.252 | ns |
| Epinephrine | Non-Smokers | Cigarette Smokers | 25 | 27 | 1.982228896 | 40.30247617 | 0.0543 | . |
| Fibrinogen | Non-Smokers | Cigarette Smokers | 30 | 33 | 0.882278397 | 56.84612112 | 0.381 | ns |
| HDL | Non-Smokers | Cigarette Smokers | 30 | 33 | -0.613567152 | 48.33430562 | 0.542 | ns |
| Hba1c | Non-Smokers | Cigarette Smokers | 30 | 33 | -0.104873113 | 60.97693078 | 0.917 | ns |
| Noradrenaline | Non-Smokers | Cigarette Smokers | 30 | 33 | 0.151264277 | 60.94805852 | 0.88 | ns |
