# Supplementary material for: A novel approach for measuring allostatic load highlights differences in stress burdens due to race, sex and smoking status
Source: PLoS One. 2025 Jun 2;20(5):e0323788. doi: 10.1371/journal.pone.0323788 (PMC12129187; doi:10.1371/journal.pone.0323788)
Supplement: S4 Table — Results are shown for acute and allostatic load scores calculated from weights derived from both the two- and three- blood pressure classes input into the ordinal regression model. P-values < 0.1 were considered significant. ∎ Represents significance between groups (p < 0.1), * represents significance between groups (p < 0.05), ** represents significance between groups (p < 0.01), *** represents significance between groups (p < 0.001). (DOCX) [file pone.0323788.s007.docx]

**S4 Table. T-test results for stress scores compared across groups (race, sex and smoking status).** Results are shown for acute and allostatic load scores calculated from weights derived from both the two- and three- blood pressure classes input into the ordinal regression model. P-values < 0.1 were considered significant. ^∎^ Represents significance between groups (p < 0.1), * represents significance between groups (p < 0.05), ** represents significance between groups (p<0.01), *** represents significance between groups (p <0.001).

| **Outcome** | **BP Class** | **Race** | | **Sex** | | **Smoking Status** | |
| --- | --- | --- | --- | --- | --- | --- | --- |
|  |  | Difference | P Value | Difference | P Value | Difference | P Value |
| **Acute Stress Score** | Two | -0.023772136 | 0.475002389 | -0.002160694 | 0.950005405 | -0.069647106 | 0.0383261191* |
| **Acute Stress Score** | Three | -0.02176105 | 0.535530302 | -0.023939647 | 0.544816451 | -0.074807146 | 0.0347149216* |
| **Secondary Mediator Score** |  | 0.157807213 | 0.009811123** | 0.032015395 | 0.592653464 | -0.054605957 | 0.38950558 |
| **Allostatic Load Score** | Two | 0.134035077 | 0.0702987332∎ | 0.029854701 | 0.672895811 | -0.124253064 | 0.101005466 |
| **Allostatic Load Score** | Three | 0.136046163 | 0.0684592051∎ | 0.008075748 | 0.910871276 | -0.129413103 | 0.0897146238∎ |
