# Supplementary material for: A novel approach for measuring allostatic load highlights differences in stress burdens due to race, sex and smoking status
Source: PLoS One. 2025 Jun 2;20(5):e0323788. doi: 10.1371/journal.pone.0323788 (PMC12129187; doi:10.1371/journal.pone.0323788)
Supplement: S5 Table — ∎Represents significance between group (P < 0.1), * Represents significance between groups (p < 0.05), ** represents significance between groups (p < 0.01), *** represents significance between groups (p < 0.001), **** represents significance between groups (p < 0.0001). (DOCX) [file pone.0323788.s008.docx]

| **Outcome** | **Blood Pressure Class** | **Race & Sex** | |  | **Smoking Status & Race** | |  | **Sex & Smoking Status** | |
| --- | --- | --- | --- | --- | --- | --- | --- | --- | --- |
|  |  | **F** | **p** |  | **F** | **p** |  | **F** | **p** |
| Acute Stress Score | Two | 0.59463961 | 0.620995718 |  | 1.883443186 | 0.14221349 |  | 1.940532856 | 0.132873926 |
| Acute Stress Score | Three | 1.882099247 | 0.142440984 |  | 1.808297461 | 0.155499684 |  | 2.174948162 | 0.100489207 |
| Secondary Mediator Score |  | 4.54618574 | 0.006214545****** |  | 3.728746819 | 0.015972625* |  | 0.47595192 | 0.700222307 |
| Allostatic Load Score | Two | 3.349905249 | 0.0249008117***** |  | 3.381731883 | 0.02398564074***** |  | 1.319594354 | 0.276563713 |
| Allostatic Load Score | Three | 4.452676822 | 0.0069159285** |  | 3.376518459 | 0.02413317391***** |  | 1.202270793 | 0.316869384 |

**S5 Table. Two-way ANOVA data comparing groups by race, sex and smoking status. ^∎^**Represents significance between group (P < 0.1), * Represents significance between groups (p < 0.05), ** represents significance between groups (p < 0.01), *** represents significance between groups (p <0.001), **** represents significance between groups (p < 0.0001).
