# Supplementary material for: A novel approach for measuring allostatic load highlights differences in stress burdens due to race, sex and smoking status
Source: PLoS One. 2025 Jun 2;20(5):e0323788. doi: 10.1371/journal.pone.0323788 (PMC12129187; doi:10.1371/journal.pone.0323788)
Supplement: S6 Table — ∎Represents significance between group (Padj < 0.1), * Represents significance between groups (Padj < 0.05), ** represents significance between groups (Padj < 0.01), *** represents significance between groups (Padj < 0.001), **** represents significance between groups (Padj < 0.0001). (DOCX) [file pone.0323788.s009.docx]

**S6 Table. Pairwise t-test post-hoc test results assessing differences in stratified groups. ^∎^**Represents significance between group (P < 0.1), * Represents significance between groups (p < 0.05), ** represents significance between groups (p < 0.01), *** represents significance between groups (p <0.001), **** represents significance between groups (p < 0.0001).

| **Outcome** | **Blood Pressure Class** |  | **Race & Sex** | | | |  | **Smoking Status & Race** | | | |  | **Sex & Smoking Status** | | | | |
| --- | --- | --- | --- | --- | --- | --- | --- | --- | --- | --- | --- | --- | --- | --- | --- | --- | --- |
|  |  |  | Variables | Difference | P Value | P Adj |  | Variables | Difference | P Value | P Adj |  | Variables | Difference | P Value | P Adj |  |
| Acute Stress Score | Two |  | B, M x B, F | 0.038719178 | 0.440287884 | 0.600124968 |  | CS, W x CS, B | -0.045444929 | 0.314936746 | 0.419622056 |  | F, NS x F, CS | -0.096402478 | 0.022437417 | 0.1346245 |  |
| Acute Stress Score | Two |  | W, F x B, F | 0.004906304 | 0.907361673 | 0.907361673 |  | NS, B x CS, B | -0.095489872 | 0.044275009 | 0.160468369 |  | M, CS x F, CS | -0.047550856 | 0.292599284 | 0.438898926 |  |
| Acute Stress Score | Two |  | W, M x B, F | -0.034932035 | 0.474000232 | 0.600124968 |  | NS, W x CS, B | -0.088348259 | 0.053489456 | 0.160468369 |  | M, NS x F, CS | -0.080543575 | 0.168215798 | 0.438898926 |  |
| Acute Stress Score | Two |  | W, F x B, M | -0.033812873 | 0.50010414 | 0.600124968 |  | NS, B x CS, W | -0.050044942 | 0.292419568 | 0.419622056 |  | M, CS x F, NS | 0.048851621 | 0.24779145 | 0.438898926 |  |
| Acute Stress Score | Two |  | W, M x B, M | -0.073651212 | 0.188946073 | 0.600124968 |  | NS, W x CS,W | -0.04290333 | 0.349685047 | 0.419622056 |  | M, NS x F, NS | 0.015858903 | 0.776047704 | 0.776047704 |  |
| Acute Stress Score | Two |  | W, M x W, F | -0.039838339 | 0.414492603 | 0.600124968 |  | NS, W x NS:B | 0.007141613 | 0.880021771 | 0.880021771 |  | M, NS x M, CS | -0.032992718 | 0.573317654 | 0.687981185 |  |
|  |  |  |  |  |  |  |  |  |  |  |  |  |  |  |  |  |  |
| Acute Stress Score | Three |  | B, M x B, F | 0.056567383 | 0.271342505 | 0.407013757 |  | CS, W x CS, B | -0.038458951 | 0.419952594 | 0.503943113 |  | F, NS x F, CS | -0.087795035 | 0.046221359 | 0.138664076 |  |
| Acute Stress Score | Three |  | W, F x B, F | 0.035517874 | 0.411232618 | 0.493479141 |  | NS, B x CS, B | -0.095692534 | 0.055889522 | 0.174419888 |  | M, CS x F, CS | -0.048674105 | 0.304131938 | 0.437596873 |  |
| Acute Stress Score | Three |  | W, M x B, F | -0.063699796 | 0.203671825 | 0.407013757 |  | NS, W x CS, B | -0.091495114 | 0.058139963 | 0.174419888 |  | M, NS x F, CS | -0.13327365 | 0.031624895 | 0.138664076 |  |
| Acute Stress Score | Three |  | W, F x B, M | -0.02104951 | 0.680968311 | 0.680968311 |  | NS, B x CS, W | -0.057233582 | 0.254627638 | 0.411480622 |  | M, CS x F, NS | 0.03912093 | 0.376300278 | 0.437596873 |  |
| Acute Stress Score | Three |  | W, M x B, M | -0.12026718 | 0.037962222 | 0.149622593 |  | NS, W x CS,W | -0.053036163 | 0.274320414 | 0.411480622 |  | M, NS x F, NS | -0.045478614 | 0.437596873 | 0.437596873 |  |
| Acute Stress Score | Three |  | W, M x W, F | -0.09921767 | 0.049874198 | 0.149622593 |  | NS, W x NS:B | 0.004197419 | 0.933050088 | 0.933050088 |  | M, NS x M, CS | -0.084599544 | 0.17129882 | 0.34259764 |  |
|  |  |  |  |  |  |  |  |  |  |  |  |  |  |  |  |  |  |
| Secondary Mediator Score |  |  | B, M x B, F | 0.175911178 | 0.043529991 | 0.130589972 |  | CS, W x CS, B | 0.069623141 | 0.390578792 | 0.468694551 |  | F, NS x F, CS | -0.085459929 | 0.28773356 | 0.897250183 |  |
| Secondary Mediator Score |  |  | W, F x B, F | 0.26177596 | 0.000568092 | 0.00340855** |  | NS, B x CS, B | -0.160319173 | 0.059415696 | 0.118831393 |  | M, CS x F, CS | -0.023612453 | 0.78643083 | 0.983709967 |  |
| Secondary Mediator Score |  |  | W, M x B, F | 0.150979556 | 0.073809247 | 0.147618494 |  | NS, W x CS, B | 0.101186871 | 0.213688681 | 0.320533022 |  | M, NS x F, CS | -0.00229346 | 0.983709967 | 0.983709967 |  |
| Secondary Mediator Score |  |  | W, F x B, M | 0.085864782 | 0.318074484 | 0.381689381 |  | NS, B x CS, W | -0.229942314 | 0.008589729 | 0.025769186* |  | M, CS x F, NS | 0.061847476 | 0.448625092 | 0.897250183 |  |
| Secondary Mediator Score |  |  | W, M x B, M | -0.024931622 | 0.793524059 | 0.793524059 |  | NS, W x CS,W | 0.03156373 | 0.700657589 | 0.700657589 |  | M, NS x F, NS | 0.083166469 | 0.442278953 | 0.897250183 |  |
| Secondary Mediator Score |  |  | W, M x W, F | -0.110796404 | 0.18677088 | 0.28015632 |  | NS, W x NS:B | 0.261506044 | 0.003033896 | 0.018203374* |  | M, NS x M, CS | 0.021318993 | 0.850828381 | 0.983709967 |  |
|  |  |  |  |  |  |  |  |  |  |  |  |  |  |  |  |  |  |
| Allostatic Load Score | Two |  | B, M x B, F | 0.214630356 | 0.044578674 | 0.133736023 |  | CS, W x CS, B | 0.024178212 | 0.803966845 | 0.908672819 |  | F, NS x F, CS | -0.181862407 | 0.056071362 | 0.336428174 |  |
| Allostatic Load Score | Two |  | W, F x B, F | 0.266682264 | 0.00365684 | 0.02194104* |  | NS, B x CS, B | -0.255809045 | 0.013528478 | 0.0270569564* |  | M, CS x F, CS | -0.07116331 | 0.486512294 | 0.635617262 |  |
| Allostatic Load Score | Two |  | W, M x B, F | 0.116047521 | 0.258573159 | 0.387859739 |  | NS, W x CS, B | 0.012838612 | 0.895120581 | 0.908672819 |  | M, NS x F, CS | -0.082837035 | 0.529681051 | 0.635617262 |  |
| Allostatic Load Score | Two |  | W, F x B, M | 0.052051909 | 0.620519059 | 0.620519059 |  | NS, B x CS, W | -0.279987257 | 0.007939426 | 0.0270569564* |  | M, CS x F, NS | 0.110699097 | 0.248502654 | 0.635617262 |  |
| Allostatic Load Score | Two |  | W, M x B, M | -0.098582834 | 0.400014134 | 0.48001696 |  | NS, W x CS,W | -0.0113396 | 0.908672819 | 0.908672819 |  | M, NS x F, NS | 0.099025372 | 0.434847906 | 0.635617262 |  |
| Allostatic Load Score | Two |  | W, M x W, F | -0.150634743 | 0.143987267 | 0.287974534 |  | NS, W x NS:B | 0.268647657 | 0.010680207 | 0.027056956* |  | M, NS x M, CS | -0.011673725 | 0.929941384 | 0.929941384 |  |
|  |  |  |  |  |  |  |  |  |  |  |  |  |  |  |  |  |  |
| Allostatic Load Score | Three |  | B, M x B, F | 0.232478561 | 0.027724839 | 0.08075631∎ |  | CS, W x CS, B | 0.03116419 | 0.751074867 | 0.921383363 |  | F, NS x F, CS | -0.173254964 | 0.071361563 | 0.428169376 |  |
| Allostatic Load Score | Three |  | W, F x B, F | 0.297293834 | 0.001120737 | 0.00672442** |  | NS, B x CS, B | -0.256011707 | 0.014211341 | 0.0284226819* |  | M, CS x F, CS | -0.072286559 | 0.484504283 | 0.726756425 |  |
| Allostatic Load Score | Three |  | W, M x B, F | 0.08727976 | 0.387235976 | 0.464683172 |  | NS, W x CS, B | 0.009691756 | 0.921383363 | 0.921383363 |  | M, NS x F, CS | -0.135567109 | 0.31033769 | 0.62067538 |  |
| Allostatic Load Score | Three |  | W, F x B, M | 0.064815273 | 0.531611346 | 0.531611346 |  | NS, B x CS, W | -0.287175896 | 0.006987084 | 0.0284226819* |  | M, CS x F, NS | 0.100968406 | 0.297367548 | 0.62067538 |  |
| Allostatic Load Score | Three |  | W, M x B, M | -0.145198802 | 0.209897609 | 0.314846414 |  | NS, W x CS,W | -0.021472433 | 0.829470317 | 0.921383363 |  | M, NS x F, NS | 0.037687855 | 0.768296323 | 0.768296323 |  |
| Allostatic Load Score | Three |  | W, M x W, F | -0.210014074 | 0.04037816 | 0.0807563 ∎ |  | NS, W x NS:B | 0.265703463 | 0.012190783 | 0.0284226819* |  | M, NS x M, CS | -0.063280551 | 0.637695524 | 0.765234629 |  |
