# Supplementary material for: A novel approach for measuring allostatic load highlights differences in stress burdens due to race, sex and smoking status
Source: PLoS One. 2025 Jun 2;20(5):e0323788. doi: 10.1371/journal.pone.0323788 (PMC12129187; doi:10.1371/journal.pone.0323788)
Supplement: S7 Table — The table includes serum biomarker concentrations measured using ELISA and associated demographic information. (DOCX) [file pone.0323788.s010.docx]

**S7 Table. Raw data for allostatic load biomarkers.** The table includes serum biomarker concentrations measured using ELISA and associated demographic information.

| **Subject_ID** | **Cortisol (ng/mL)** | **Noradrenaline (ng/mL)** | **Epinephrine (ng/mL)** | **HbA1c (mg/mL)** | **Fibrinogen (µg/mL)** | **CRP (µg/mL)** | **HDL (mg/mL)** |
| --- | --- | --- | --- | --- | --- | --- | --- |
| CS_1 | 140.304 | 2638.625 | 5.405 | 3000.829 | 944805.237 | 55598.4537 | 2342.104 |
| CS_2 | 246.542 | 513.244 | 5.994 | 1998.931 | 559138.534 | 1406410.93 | 4901.3985 |
| CS_3 | 50.878 | 518.569 | 4.347 | 1296.188 | 832811.673 | 5849888.15 | 2256.1675 |
| CS_4 | 137.387 | 1089.961 | 4.959 | 1213.764 | 750575.05 | 36677139.3 | 7471.9295 |
| CS_5 | 116.602 | 6214.28 | n.a. | 8901.778 | 1106446.96 | 896782.493 | 1549.526 |
| CS_6 | 54.03 | 1123.025 | 6.566 | 921.903 | 530825.467 | 210382.666 | 1770.204 |
| CS_7 | 48.235 | 478.157 | 5.406 | 1481.45 | 745863.327 | 398588.87 | 1182.1405 |
| CS_8 | 131.446 | 3571.192 | n.a. | 13346.328 | 540446.084 | 483245.229 | 1352.725 |
| CS_9 | 162.791 | 2154.116 | 5.122 | 383.474 | 884242.874 | 2137266.82 | 1570.423 |
| CS_10 | 89.446 | 5745.697 | 6.495 | 20334.869 | 1177711.46 | 2863647.26 | 678.193 |
| CS_11 | 71.309 | 1402.178 | n.a. | 199.15 | 355916.019 | 77646.9783 | 1638.452 |
| CS_12 | 100.023 | 3920.142 | 4.843 | 9529.201 | 2570534.26 | 208024.677 | 3487.302 |
| CS_13 | 68.341 | 561.648 | 4.221 | 171.814 | 749161.433 | 4750067.98 | 2103.0105 |
| CS_14 | 58.318 | 2617.326 | 5.394 | 611.658 | 290255.078 | 3478791.45 | 1945.3195 |
| CS_15 | 45.239 | 355.228 | 8.817 | 610.929 | 378887.002 | 5037985.01 | 1468.4135 |
| CS_16 | 74.484 | 3571.192 | 10.947 | 9741.94 | 386630.497 | 1296295.82 | 1164.451 |
| CS_17 | 163.209 | 2486.259 | 9.02 | 13243.567 | 934662.933 | 845842.458 | 2309.097 |
| CS_18 | 8.615 | 1333.108 | 7.161 | 409.45 | 992917.633 | 4158242.31 | 2216.1165 |
| CS_19 | 12.477 | 1203.403 | n.a. | 878.49 | 800601.933 | 25307843.8 | 1648.697 |
| CS_20 | 125.198 | 2671.216 | 3.724 | 27388.5267 | 726553.321 | 3968823.1 | 2356.5275 |
| CS_21 | 109.448 | 2116.57 | 6.167 | 22539.139 | 899081.334 | 6018344.51 | 813.817 |
| CS_22 | 68.909 | 2493.733 | 8.476 | 18983.376 | 714782.873 | 1509256.84 | 1065.085 |
| CS_23 | 65.721 | 1848.498 | 4.517 | 22177.338 | 1729657.36 | 1858597.83 | 1460.5 |
| CS_24 | 110.232 | 4635.515 | 4.994 | 23200.14 | 1228578.68 | 4368597.99 | 1395.579 |
| CS_25 | 71.477 | 3429.444 | n.a. | 989.243 | 318988.666 | 2316134.67 | 2616.292 |
| CS_26 | 94.08 | 406.851 | n.a. | 10037.913 | 1453148.06 | 1542552.36 | 661.033 |
| CS_27 | 80.109 | 1882.72 | 3.964 | 15099.842 | 479839.559 | 1402123.8 | 1079.275 |
| CS_28 | 58.67 | 930.578 | 7.482 | 11638.164 | 733616.721 | 1782834.01 | 850.757 |
| CS_29 | 74.571 | 976.177 | 7.334 | 14044.853 | 950126.299 | 2257170.73 | 1564.179 |
| CS_30 | 64.56 | 4034.549 | 5.63 | 20676.633 | 1369731.75 | 902047.715 | 1422.76 |
| CS_31 | 90.608 | 6712.651 | 4.653 | 27408.118 | 1911426.32 | 1044220.74 | 3840.475 |
| CS_32 | 91.571 | 2022.624 | 4.98 | 19463.748 | 2586600.94 | 18215984.6 | 1719.756 |
| CS_33 | 67.139 | 637.958 | 5.465 | 12489.422 | 827119.154 | 12515521.2 | 1145.426 |
| NS_34 | 10.691 | 1132.377 | 7.261 | 976.584 | 820956.567 | 111256.605 | 1836.224 |
| NS_35 | 13.026 | 3438.432 | 6.127 | 27380.544 | 699246.693 | 566829.329 | 2203.527 |
| NS_36 | 68.341 | 2421.451 | 5.522 | 426.782 | 477878.71 | 674040.75 | 2497.084 |
| NS_37 | 106.118 | 3939.929 | 7.893 | 13432.363 | 1885217.24 | 1003162.19 | 1456.774 |
| NS_38 | 292.804 | 6659.208 | 7.33 | 13022.708 | 1244852.07 | 801701.769 | 1082.44 |
| NS_39 | 81.722 | 1328.501 | 7.524 | 13570.777 | 2340365.06 | 333720.445 | 597.406 |
| NS_40 | 59.594 | 4154.249 | 5.88 | 18681.789 | 877076.061 | 85325.9711 | 1349.759 |
| NS_41 | 246.178 | 5745.697 | 8.936 | 12276.953 | 1255399.71 | 31912764.4 | 2014.736 |
| NS_42 | 86.759 | 1045.947 | n.a. | 1787.173 | 997806.794 | 1328649.15 | 3027.9675 |
| NS_43 | 85.046 | 1499.694 | 7.336 | 821.853 | 457686.857 | 479849.044 | 1781.3585 |
| NS_44 | 294.683 | 828.987 | 5.159 | 490.276 | 368495.127 | 3270808.3 | 2920.244 |
| NS_45 | 161.13 | 1147.221 | 3.829 | 253.56 | 544767.372 | 3593039.23 | 2758.6205 |
| NS_46 | 71.057 | 1144.009 | 6.921 | 1531.42 | 543807.499 | 5561785.4 | 2118.0695 |
| NS_47 | 92.002 | 1170.196 | 7.409 | 167.856 | 391256.911 | 624488.844 | 1737.316 |
| NS_48 | 4.3075 | 1282.121 | 7.149 | 896.473 | 836608.891 | 2813744.73 | 2479.4805 |
| NS_49 | 112.759 | 4610.083 | 6.23 | 20562.294 | 1635063 | 2370375.87 | 1053.588 |
| NS_50 | 150.65 | 4370.318 | 7.463 | 16738.233 | 3413228.02 | 7755454.1 | 1771.832 |
| NS_51 | 70.224 | 2205.902 | 18.576 | 174.173 | 399446.865 | 201656.664 | 1907.8705 |
| NS_52 | 65.177 | 1499.694 | n.a. | 557.1 | 1072870.01 | 2381721.28 | 2927.837 |
| NS_53 | 137.048 | 428.669 | 7.795 | 20425.639 | 1100912.08 | 45767158.6 | 2262.79 |
| NS_54 | 96.661 | 2508.815 | n.a. | 13415.127 | 691242.249 | 256761.89 | 1385.516 |
| NS_55 | 88.818 | 907.385 | 3.842 | 448.513 | 984131.25 | 7252398.14 | 2639.136 |
| NS_56 | 109.839 | 1520.311 | n.a. | 14187.385 | 1045044.33 | 6516543.96 | 890.429 |
| NS_57 | 116.183 | 937.713 | 9.262 | 15722.74 | 1633222.34 | 4517125.57 | 1518.944 |
| NS_58 | 92.544 | 2122.037 | 5.655 | 11906.903 | 862290.087 | 98303.1652 | 1357.09 |
| NS_59 | 104.002 | 1360.782 | 6.193 | 21512.253 | 1027264.24 | 27820113.7 | 1158.884 |
| NS_60 | 93.969 | 1856.942 | n.a. | 10691.952 | 876598.566 | 15608614.7 | 1261.489 |
| NS_61 | 109.578 | 4560.047 | 8.111 | 7531.294 | 2277012.61 | 17340713.2 | 2226.878 |
| NS_62 | 70.89 | 3411.395 | 5.844 | 21022.158 | 1303893.22 | 45143780.8 | 1026.427 |
| NS_63 | 109.448 | 2032.663 | 8.646 | 16401.634 | 726082.465 | 10552214.7 | 1020.604 |
