# Supplementary material for: A novel approach for measuring allostatic load highlights differences in stress burdens due to race, sex and smoking status
Source: PLoS One. 2025 Jun 2;20(5):e0323788. doi: 10.1371/journal.pone.0323788 (PMC12129187; doi:10.1371/journal.pone.0323788)
Supplement: S8 Table — Scores were calculated using weighted formulas based on two- and three-class ordinal regression models. Secondary mediators did not have a physiological proxy to calculate weighted scores and therefore only have a single score reported. (DOCX) [file pone.0323788.s011.docx]

**S8 Table. Raw data for acute, secondary and allostatic load scores derived from serum biomarkers.** Scores were calculated using weighted formulas based on two- and three-class ordinal regression models. Secondary mediators did not have a physiological proxy to calculate weighted scores and therefore only have a single score reported.

| Subject_ID | Acute_Stress_Two | Acute_Stress_Three | Secondary_Mediators | Allostatic_Load_Two | Allostatic_Load_Three |
| --- | --- | --- | --- | --- | --- |
| CS _5 | 0.251690452 | 0.16756925 | 0.143841059 | 0.395531511 | 0.31141031 |
| CS_ 2 | 0.500356257 | 0.346733232 | -0.111592548 | 0.388763709 | 0.235140684 |
| CS_ 8 | 0.265981709 | 0.164310082 | 0.135876629 | 0.401858338 | 0.300186711 |
| CS_ 3 | 0.128145769 | 0.092809693 | 0.037497839 | 0.165643608 | 0.130307532 |
| CS_10 | 0.319081203 | 0.301783431 | 0.307339542 | 0.626420745 | 0.609122973 |
| NS_62 | 0.249409379 | 0.231926681 | 0.532113647 | 0.781523026 | 0.764040328 |
| CS_ 1 | 0.340905835 | 0.25578387 | 0.022740537 | 0.363646372 | 0.278524407 |
| NS_61 | 0.415399912 | 0.404968449 | 0.30283709 | 0.718237002 | 0.707805539 |
| NS_59 | 0.312809773 | 0.266738418 | 0.42797063 | 0.740780403 | 0.694709048 |
| NS_57 | 0.454332081 | 0.445903789 | 0.280154839 | 0.73448692 | 0.726058627 |
| NS_56 | 0.223117676 | 0.133122591 | 0.260452805 | 0.48357048 | 0.393575396 |
| CS_14 | 0.202527969 | 0.186490332 | -0.032701604 | 0.169826365 | 0.153788728 |
| NS_55 | 0.173973819 | 0.095405808 | 0.035002218 | 0.208976037 | 0.130408026 |
| CS_ 4 | 0.308229169 | 0.212367413 | 0.025408921 | 0.33363809 | 0.237776334 |
| NS_ 53 | 0.427354769 | 0.378223237 | 0.455527001 | 0.88288177 | 0.833750238 |
| NS_50 | 0.452409861 | 0.399168668 | 0.42064724 | 0.873057101 | 0.819815908 |
| CS_ 6 | 0.239303356 | 0.243366483 | -0.01897933 | 0.220324026 | 0.224387152 |
| CS_7 | 0.173114893 | 0.160090231 | 0.039356223 | 0.212471116 | 0.199446454 |
| NS_47 | 0.343200679 | 0.327047247 | -0.039394148 | 0.303806531 | 0.287653098 |
| CS_16 | 0.45621341 | 0.514928016 | 0.100666142 | 0.556879552 | 0.615594158 |
| NS_39 | 0.33093899 | 0.326121469 | 0.328532058 | 0.659471047 | 0.654653526 |
| CS_17 | 0.524216569 | 0.481135955 | 0.135054473 | 0.659271042 | 0.616190428 |
| CS_11 | 0.155650105 | 0.098201831 | -0.042765726 | 0.112884378 | 0.055436105 |
| NS_37 | 0.398786482 | 0.386326725 | 0.256813015 | 0.655599497 | 0.64313974 |
| CS_33 | 0.212232004 | 0.183002038 | 0.251081735 | 0.463313739 | 0.434083773 |
| CS_9 | 0.358766772 | 0.250377071 | 0.033961067 | 0.392727839 | 0.284338139 |
| CS_12 | 0.255838022 | 0.19586505 | 0.178900621 | 0.434738642 | 0.374765671 |
| CS_27 | 0.169970463 | 0.104648562 | 0.164930893 | 0.334901356 | 0.269579455 |
| CS_22 | 0.351459337 | 0.376669338 | 0.222933932 | 0.57439327 | 0.59960327 |
| CS_18 | 0.173051529 | 0.234301374 | 0.031115511 | 0.20416704 | 0.265416885 |
| CS_29 | 0.30862925 | 0.30592522 | 0.187146093 | 0.495775343 | 0.493071313 |
| CS_20 | 0.236319985 | 0.130760554 | 0.244483657 | 0.480803643 | 0.375244212 |
| CS_32 | 0.240015436 | 0.184698049 | 0.458062465 | 0.6980779 | 0.642760514 |
| NS_36 | 0.22603037 | 0.202445182 | -0.058700209 | 0.167330162 | 0.143744973 |
| CS_28 | 0.285353617 | 0.299535463 | 0.174951268 | 0.460304885 | 0.474486731 |
| CS_23 | 0.1712815 | 0.129692422 | 0.321605396 | 0.492886895 | 0.451297818 |
| NS_38 | 0.636309301 | 0.494196501 | 0.216868411 | 0.853177711 | 0.711064912 |
| NS_40 | 0.233145731 | 0.228567242 | 0.211921931 | 0.445067662 | 0.440489173 |
| CS_25 | 0.165328681 | 0.113629687 | -0.061442032 | 0.103886649 | 0.052187655 |
| CS_31 | 0.240200643 | 0.190604507 | 0.269140625 | 0.509341267 | 0.459745132 |
| NS_34 | 0.180673447 | 0.240616599 | 0.007838828 | 0.188512275 | 0.248455427 |
| NS_35 | 0.147972894 | 0.192961783 | 0.222487888 | 0.370460782 | 0.415449671 |
| NS_41 | 0.64267518 | 0.551117975 | 0.352899329 | 0.995574509 | 0.904017303 |
| CS_26 | 0.190514212 | 0.109726572 | 0.233909359 | 0.424423572 | 0.343635932 |
| NS_49 | 0.342585519 | 0.298730417 | 0.325371271 | 0.66795679 | 0.624101688 |
| NS_51 | 0.668221924 | 0.816354599 | -0.049300758 | 0.618921165 | 0.767053841 |
| NS_43 | 0.329921377 | 0.31969085 | -0.026586683 | 0.303334694 | 0.293104167 |
| NS_44 | 0.519995032 | 0.324376606 | -0.067305262 | 0.45268977 | 0.257071344 |
| CS_24 | 0.282459018 | 0.218513783 | 0.308663503 | 0.591122521 | 0.527177286 |
| CS_30 | 0.230651072 | 0.2167031 | 0.275234029 | 0.5058851 | 0.491937128 |
| NS_42 | 0.180279757 | 0.10758236 | -0.004799689 | 0.175480068 | 0.10278267 |
| NS_45 | 0.288402407 | 0.153119066 | -0.042321503 | 0.246080904 | 0.110797563 |
| NS_46 | 0.286023955 | 0.280267615 | 0.014717218 | 0.300741173 | 0.294984833 |
| NS_58 | 0.273326403 | 0.229812786 | 0.152438455 | 0.425764859 | 0.382251241 |
| CS_21 | 0.324311147 | 0.275633846 | 0.31351465 | 0.637825797 | 0.589148496 |
| NS_63 | 0.425676761 | 0.417027533 | 0.268729771 | 0.694406533 | 0.685757304 |
| NS_48 | 0.16278846 | 0.228454941 | 0.001522672 | 0.164311132 | 0.229977612 |
| CS_19 | 0.036984124 | 0.036661678 | 0.171186768 | 0.208170892 | 0.207848446 |
| NS_52 | 0.145699275 | 0.094470626 | -0.001613631 | 0.144085644 | 0.092856994 |
| CS_15 | 0.309066217 | 0.354902076 | 0.010200377 | 0.319266594 | 0.365102453 |
| NS_54 | 0.204038573 | 0.127246939 | 0.148942264 | 0.352980837 | 0.276189203 |
| NS_ 60 | 0.198126571 | 0.122368075 | 0.252243472 | 0.450370043 | 0.374611547 |
| CS_13 | 0.154784758 | 0.100740833 | 0.01333274 | 0.168117498 | 0.114073573 |
